# Critical review of the safety assessment of titanium dioxide additives in food

**DOI:** 10.1186/s12951-018-0376-8

**Published:** 2018-06-01

**Authors:** Hans Christian Winkler, Tina Notter, Urs Meyer, Hanspeter Naegeli

**Affiliations:** 1 Institute of Food, Nutrition and Health, Department of Health Sciences and Technology, ETH Zurich, Schmelzbergstrasse 7, 8092 Zurich, Switzerland; 2 Institute of Pharmacology and Toxicology, University of Zurich-Vetsuisse, Winterthurerstrasse 260, 8057 Zurich, Switzerland

**Keywords:** Anatase, Cancer, Corona, Dendritic cells, Food additives, Food safety, Food toxicology, Innate immunity, Lymphoid tissue, Rutile

## Abstract

Nanomaterial engineering provides an important technological advance that offers substantial benefits for applications not only in the production and processing, but also in the packaging and storage of food. An expanding commercialization of nanomaterials as part of the modern diet will substantially increase their oral intake worldwide. While the risk of particle inhalation received much attention, gaps of knowledge exist regarding possible adverse health effects due to gastrointestinal exposure. This problem is highlighted by pigment-grade titanium dioxide (TiO_2_), which confers a white color and increased opacity with an optimal particle diameter of 200–300 nm. However, size distribution analyses showed that batches of food-grade TiO_2_ always comprise a nano-sized fraction as inevitable byproduct of the manufacturing processes. Submicron-sized TiO_2_ particles, in Europe listed as E 171, are widely used as a food additive although the relevant risk assessment has never been satisfactorily completed. For example, it is not possible to derive a safe daily intake of TiO_2_ from the available long-term feeding studies in rodents. Also, the use of TiO_2_ particles in the food sector leads to highest exposures in children, but only few studies address the vulnerability of this particular age group. Extrapolation of animal studies to humans is also problematic due to knowledge gaps as to local gastrointestinal effects of TiO_2_ particles, primarily on the mucosa and the gut-associated lymphoid system. Tissue distributions after oral administration of TiO_2_ differ from other exposure routes, thus limiting the relevance of data obtained from inhalation or parenteral injections. Such difficulties and uncertainties emerging in the retrospective assessment of TiO_2_ particles exemplify the need for a fit-to-purpose data requirement for the future evaluation of novel nano-sized or submicron-sized particles added deliberately to food.

## Background

Potential applications of recent nanomaterial developments in the food sector include, for example, nano-sized coatings of packaging materials to protect from mechanical damage or microbial contamination, thereby extending the shelf life. Nano-sized additives may also be deliberately incorporated in food to optimize properties such as taste, sensation, color, texture or consistency. Nanomaterials may be employed to supplement food with vitamins in a highly bioavailable form and could contribute to prevent nutritional iron deficiency and anemia, affecting nearly 2 billion people worldwide [1–3]. Nano-sized materials may further provide markers of food freshness and quality, or allow for traceability and the detection of pathogens or contaminants [4, 5]. In contrast to these novel developments, submicron-sized particles of titanium dioxide (TiO_2_) have been used in the food sector for more than 50 years as a pigment to enhance the white color and opacity of foods like coffee creamer, sauces, spreads, pastries, candies and edible ices. Also, TiO_2_ confers brightness to toothpaste and is added to enhance the flavor of non-white foods (processed fish, fruits, meat, vegetables, breakfast cereals, fermented soybean, soups and mustard) and to clear beverages (beer, cider and wine) [6–9].

Currently, the annual consumption volume of TiO_2_ particles reaches four million tons, which makes it the most widely used pigment globally [10]. In the United States (US), the Food and Drug Administration allows up to 1% by weight of TiO_2_ particles as a food colorant [11]. In the European Union (EU), TiO_2_ is an authorized food additive (listed as E 171) at *quantum satis*, meaning that no maximum level is imposed as long as the additive is used in accordance with good manufacturing practice, i.e., at a level not higher than necessary to achieve the intended scope [12]. A comparison of use levels reported by the food industry show that the highest TiO_2_ concentrations are expected in chewing gum (up to 16,000 mg/kg), food supplements delivered in a solid form (up to 12,000 mg/kg), processed nuts (up to 7000 mg/kg) and ready-to-use salads and sandwich spreads (up to 3000 mg/kg) [13]. TiO_2_ particles can, therefore, be viewed as a paradigmatic case for the safety assessment of inorganic particles employed as food additive and comprising a nano-scale fraction.

The standard risk assessment procedure with risk = hazard × exposure, which includes hazard identification, hazard characterization, exposure assessment and risk characterization, is also applicable to small inorganic particles in food. The prefix “nano” does not make a substance automatically harmful and possible adverse effects should be tested case-by-case. However, reductions in size may change the material characteristics as compared to larger particles or the same substance in solution. Nano-sized particles display an increased surface-to-mass ratio that enhances their reactivity [14, 15]. Also, nanoparticles display an increased propensity to penetrate through cell membranes thus conferring the potential for trafficking across biological barriers including the intestinal mucosa [16–18]. In principle, a nanomaterial exists in different forms, i.e., with one dimension in the nano-scale (for example nano-films), two dimensions in the nano-scale (for example nano-rods) or, as for nanoparticles, all three dimensions in the nano-scale range. A European Commission Recommendation defines nanomaterials as natural, incidental or manufactured materials, containing 50% or more of the particles, determined in a number-based size distribution, with at least one external dimension not exceeding 100 nm [19]. However, there is no scientific ground to defend such a strict size boundary in the identification of possible hazards, as one would rather expect a gradient in the capacity of eliciting adverse effects with changing particle dimensions. In any case, a final answer to the question of when a material becomes nano-sized has not been provided [20] and the above Recommendation is not yet adopted for regulatory purposes.

Until now, the health effects of TiO_2_ particles have been studied mainly with regard to their uptake by inhalation [21–23]. The International Agency for Research on Cancer (IARC) concluded that there is inadequate evidence from epidemiological studies to assess whether TiO_2_ dust causes cancer in humans, but that there is sufficient evidence for carcinogenicity in experimental animals, based on the induction of respiratory tract tumors in rats after prolonged inhalation [24, 25]. Therefore, IARC classified TiO_2_ as a Group 2B carcinogen [26]. Considering the widespread food-related uses, there is a pressing need to review the suitability of studies supporting the risk assessment of TiO_2_ particles as food additive [27]. Comprehensive reviews on this topic have been provided *inter alia* by Shi et al. [28], Heringa et al. [29] and the Scientific Panel on Food Additives and Nutrient Sources added to Food (ANS Panel) [13]. The purpose of our contribution is to focus on data gaps and uncertainties in relevant risk assessment studies covering the dietary uptake of TiO_2_ particles.

### TiO_2_ particle manufacture and their physicochemical properties

Although Ti is the ninth most abundant element in the earth’s crust, it never appears in a metallic state in nature. TiO_2_, an odorless powder with a molecular weight of 79.9 g/mol, also known as Ti(IV) oxide, constitutes the naturally occurring oxide [30, 31]. TiO_2_ minerals contain impurities such as iron, chromium, vanadium or zirconium that confer a spectrum of different colors. Manufactured TiO_2_ is, instead, a white powder commonly used as a pigment in ceramics, paints, coatings, plastics and paper due to its high refractive index. Pure TiO_2_ assembles in three crystal structures, i.e., anatase, rutile (with tetragonal coordination of Ti atoms) and brookite (with rhombohedral coordination of Ti atoms), but only anatase/rutile or mixtures of these two polymorphs are employed in food [32]. In addition, as a fourth form, amorphous TiO_2_ has been described [33]. The surface of anatase crystals is considered to be more reactive than that of rutile counterparts, as indicated by their ability to generate reactive oxygen species in aqueous solutions when irradiated with ultraviolet (UV) light [34]. Also, anatase nanoparticles display a stronger adjuvant activity than rutile nanoparticles in an allergy model based on the intranasal sensitization of mice with ovalbumin [35]. Nonetheless, the anatase form is the most frequently used in the food sector [8, 36, 37].

Food-grade TiO_2_ is manufactured from Ti minerals by either a sulfuric acid-based process, which can yield anatase, rutile or a mixture of both polymorphs depending on the reaction conditions, or a chlorine-based process yielding only the rutile form [32]. Specifications for food use include a minimum purity of 99.0%, thus allowing some contamination with arsenic, cadmium and mercury (up to 1 mg/kg), antimony (up to 2 mg/kg) or lead (up to 10 mg/kg). Also, food-grade TiO_2_ may be coated with a small proportion (no more than 2% in total) of alumina and silica to enhance technological properties, for example to improve dispersion in host matrices [32, 38]. All TiO_2_ particles are insoluble in water, organic solvents, hydrochloric acid and dilute sulfuric acid. They are highly stable to heat and remain unaffected by food processing. Also, they are not or only minimally degraded or dissolved under conditions, including low pH, which mimic the gastrointestinal milieu [39, 40]. Such indigestible particles, once released from the food matrix during their gastrointestinal transit, reach the intestinal mucosa raising the question of whether they might be prone to absorption and systemic distribution.

**Fig. 1:**
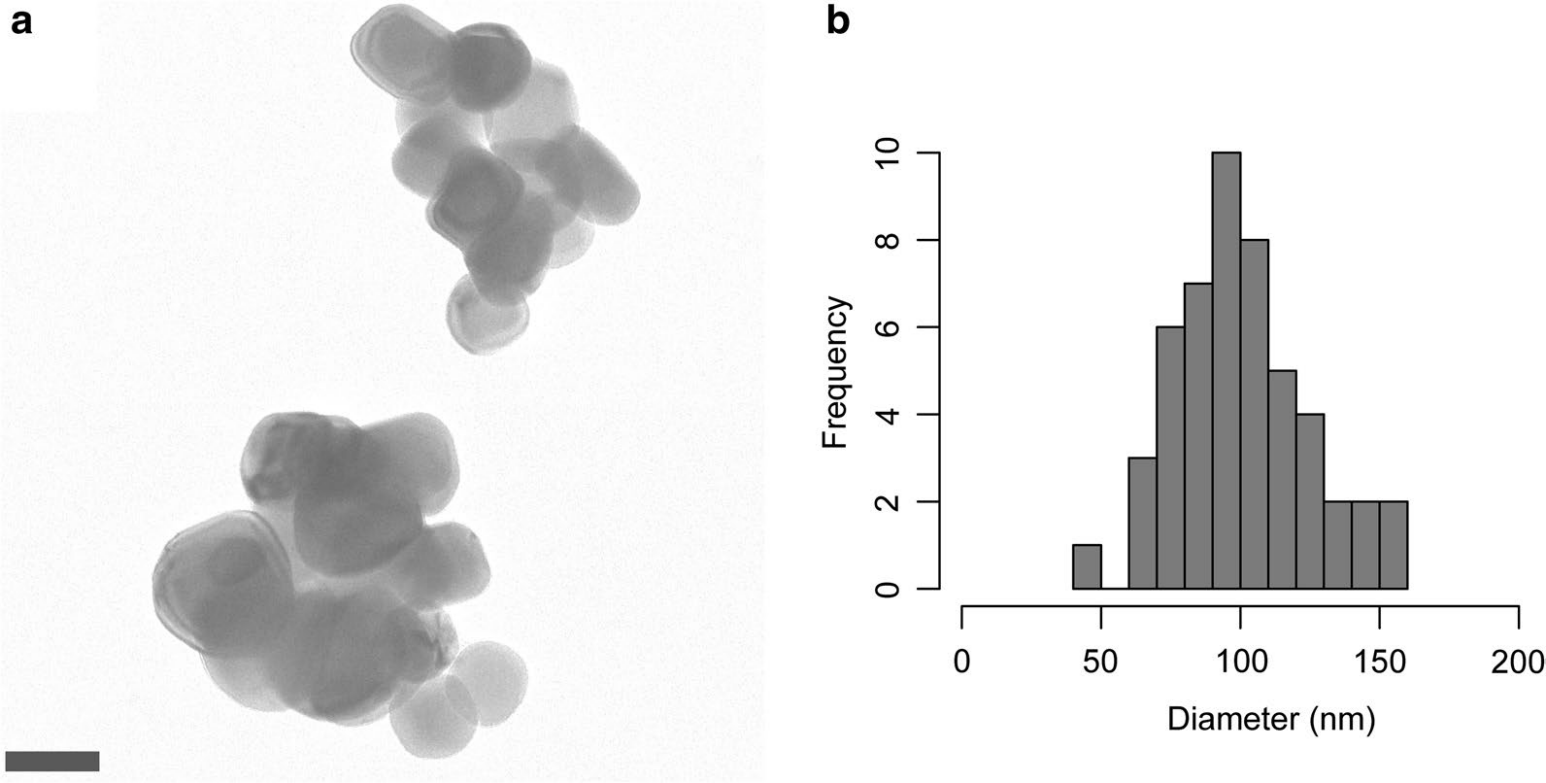
Example of food-grade TiO_2_ particles (E 171). **a** A sample of food-grade anatase dispersed in H_2_O was deposited on a copper grid coated with glow-discharged parlodion and analyzed by TEM as described [146, 147]. Scale bar, 100 nm. **b** Size distribution of the imaged food-grade TiO_2_ particles. The diameter measured as longest distance across particles is 100 ± 24 nm (mean ± standard deviation) and 54% by number of the particles have a diameter < 100 nm

Optimal light scattering is needed to achieve the desired whitening effect. Therefore, food-grade TiO_2_ ideally displays a primary particle size of approximately half the wavelength of the light to be scattered [41], i.e., half of the 400–700-nm of the visible range. Accordingly, scattering of visible light is maximized in fine particles that are 200–300 nm in diameter. Ultrafine products are instead not suited for this purpose as they become transparent when their size remains below the 100-nm threshold [42]. Such nano-sized TiO_2_ particles are often included at concentrations of up to 25% in cosmetic preparations, including lip balms and sunscreens to protect from solar light by reflecting UV radiation away from the skin [43, 44]. As a consequence of the production process there is inevitably a broad size distribution that comprises nanoparticles with a primary size below 100 nm even when the mean diameter reaches 200–300 nm. In this respect, a frequently cited size distribution is the one determined by Weir et al. [6] using transmission electron microscopy (TEM), whereby 36% of particles by number were below the threshold of 100 nm. This data was derived from a single determination with one lot of E 171 and, hence, is not representative for all TiO_2_ on the market. In a follow-up distribution analysis of five different food-grade TiO_2_ samples by TEM, nano-sized particles occured with a frequency between 17 and 35% by number [8]. Studies by scanning electron microscopy (SEM) suggested that commercial E 171 materials contain ~ 10% of particles with dimensions below 100 nm [7]. Clearly, the outcome of particle size determinations varies with the method of measurement, whereby smaller diameters are generally reported from TEM measurements compared for example to laser diffraction [13, 45]. Another relevant aspect is that, as illustrated in Fig. 1, suspended TiO_2_ particles tend to aggregate/agglomerate to form larger clusters, although a majority of the individual particles may display a primary diameter < 100 nm. The term “aggregate” designates an assembly of particles held together by covalent or metallic bonds. Instead, “agglomerates” result from weak forces like van der Waals interactions, hydrogen bonding, electrostatic attractions or adhesion by surface tensions. It is important not to equate the nanoparticle fraction measured by number with the same value by mass. The ANS Panel at the European Food Safety Authority (EFSA) proposes to use a proportion of 3.2% by mass to estimate the nano-sized fraction of E 171 for risk assessment considerations [13]. TEM analyses of TiO_2_ particles in the coatings of chewing gums revealed a nano-sized mass fraction of 4.2% on the average [45, 46]. This review only includes studies where TiO_2_ test materials have been characterized with respect to their size preferably with indications on the primary particle diameter.

### Human exposure

In the US, the dietary intake of TiO_2_ was estimated at 1–2 mg/kg body weight per day for children under the age of 10 years and 0.2–0.7 mg/kg/day for other age groups. This dietary exposure in the United Kingdom (UK) population was estimated to be 2–3 mg/kg/day for children and around 1 mg/kg/day for the other age groups [6]. The corresponding exposure values estimated for the German population are between 0.5 and 1 mg/kg/day in adults but reach ~ 2 mg/kg/day in children [47]. To obtain dietary exposures across Europe, the ANS Panel at EFSA selected food categories for which the use of TiO_2_ is authorized, and assumed that 60–80% by weight of these food items actually contain TiO_2_ as an additive. Next, the ANS Panel used the EFSA Comprehensive European Food Consumption Database and the typical TiO_2_ inclusion levels reported by industry (see background section above), as well as reported analytical results, to calculate chronic dietary exposures to TiO_2_ for different age groups. The highest values were found for children of 3–9 years where, depending on the dietary habits, the mean exposures were 0.9–8.8 mg/kg body weight per day with 95th percentiles of 2.4–30.2 mg/kg per day. Some relevant food categories (for example edible cheese rind) are not displayed in the Consumption Database and, as a consequence, could not be taken into account for the exposure estimate. Also, the contribution of the accidental swallowing of toothpaste or lip balms was not included in these calculations, possibly resulting in an underestimation of oral TiO_2_ intake. Another study employed the Dutch National Food Consumption Survey and the TiO_2_ concentrations in food products reported by industry [48]. Again, the highest exposure (median of 1.4 mg/kg body weight per day and 95th percentile of 4.9 mg/kg) was found in children 2–6 years old. A parallel study also employed the Dutch National Food Consumption Survey but used Ti and/or TiO_2_ concentrations in food products and toothpaste as reported in the literature [36]. The calculations confirmed that the highest intake (median of 0.59 mg/kg body weight per day and 95th percentile of 1.29 mg/kg) is found in children 2–6 years old. The generally elevated exposure of children is attributed to their lower body mass and disproportionately higher consumption of TiO_2_-containing products like pastries and candies [6, 13].

### Determinants of intestinal uptake

After oral exposure, foreign particles released by digestion from the food matrix encounter a layer of enterocytes, lining the intestinal tract, that are responsible for nutrient absorption. This digestive epithelium surface, in humans estimated to 30 m^2^ [49], presents a structural barrier to foreign materials that also secretes a protective layer of mucus. Indigestible particles like the ones consisting of TiO_2_ may nevertheless gain entry into the underlying *lamina propria* by penetration across or between intact enterocytes. However, the digestive mucosa is additionally defended by the gut-associated lymphoid tissue (GALT), which is arranged into lymphoid follicles that, in the small intestine, aggregate to form Peyer’s patches [50–52]. The epithelium covering this intestinal lymphoid tissue displays phagocytic microfold cells (M-cells), whose specialized function is to absorb particulates from the intestinal lumen to be forwarded to the innate immune system including dendritic cells and macrophages [53]. Intestinal dendritic cells also reach out their membrane projections across the epithelial barrier into the gut lumen to take up particulates directly [54]. Thus, TiO_2_ particle can be incorporated by cells of the innate immune system, where they persist without being substantially degraded or dissolved [16]. The local accumulation of such particles appears as pigments in the lymphoid tissue of the intestinal mucosa [51, 55–57].

### Oral bioavailability in rodents

Inhalation studies in animals converge on the finding that nano-sized TiO_2_ particles can enter, in small amounts, the systemic circulation from the alveolar epithelium and disseminate into other organs [21, 23, 58, 59]. Instead, dermal exposure studies indicated that TiO_2_ particles of any size do not penetrate the *stratum corneum* of the skin [60–63]. Less certain is the extent of intestinal absorption, but an elegant vanadium (V) radiotracer study established that the vast majority of ingested TiO_2_ nanoparticle is directly excreted in the feces. Briefly, commercial anatase particles were irradiated with a proton beam to generate a radiolabeled [^48^V]TiO_2_ fraction with a mean particle diameter of 50 nm. After demonstrating that most ^48^V ions remain associated with TiO_2_ particles, a single dose (30–80 µg/kg body weight) of this test material was administered by intraesophageal instillation (oral gavage) to Wistar-Kyoto rats [64]. Groups of animals were sacrificed 1, 4, 24 h and 7 days after gavage to assess the transfer of radioactivity from the gastrointestinal tract into the cardiovascular system and various tissues. This time course revealed that a small proportion of the applied radioactivity (only ~ 0.6%) was detected in the blood and internal organs like liver, spleen and kidneys, at 1 h after gavage. This overall proportion of systemically distributed TiO_2_ particles gradually dropped to ~ 0.05% after 7 days.

Table 1 presents an overview of relevant oral exposure studies in rodents found in the literature. A high-dose biokinetic/acute toxicity study was carried out in CD-1 mice after a single administration by oral gavage of differentially sized TiO_2_ particles (crystal structure not specified) administered at 5000 mg/kg body weight [42]. The mean diameter of the three tested particles was 25 nm, 80 nm and 155 nm. Ti concentrations in tissues were determined by inductively coupled plasma (ICP)-mass spectrometry 2 weeks after treatment. In animals that received the fine particles of 155 nm in diameter, increased Ti concentrations (~ 500 ng/g) over control levels (< 100 ng/g) were found only in the spleen. For comparison, the highest Ti level (~ 4000 ng/g) was detected in the liver of animals that received the 80-nm particles. A major deficiency of this and other bioavailability studies is that distribution measurements rely on a chemical Ti analysis and, therefore, it is not clear whether the Ti detected in fluids and tissues is due to translocation of TiO_2_ from the gastrointestinal tract in the form of particles or as solubilized material. A subsequent experiment in CD-1 mice involved the oral administration by gavage of anatase particles with mean diameters of 18 and 120 nm. The dose was 64 mg/kg body weight [65]. Increased Ti levels, measured by ICP-optical emission spectrometry, were detected in the blood, liver and pancreas, but only in animals administered the 18-nm particles. A peak Ti blood concentration of ~ 0.15 µg/ml (against a background of ~ 0.05 µg/ml) was detected 1 h after administration.

**Table 1:** Overview of oral toxicokinetic and toxicodynamic studies in rodents

Study type	Oral dose	Particle structure (mean size)	Internal exposure	Main reported effects	Source
Acute toxicity in mice	5000 mg/kg	Structure not specified (25, 80 and 155 nm)	~ 4 µg/g Ti in liver	Histopathologic findings in brain, liver and kidney	[42]
Acute toxicity in rats	Up to 5000 mg/kg	Coated rutile/anatase (73 nm)	Not examined	None	[82]
Bioavailability in rats	Up to 80 µg/kg	Radiolabeled anatase (50 nm)	Oral particle bioavailability of ~ 0.6%	None	[64]
Bioavailability in rats	5 mg/kg	Anatase and rutile (40 nm–5 µm)	None detected	None	[40]
Toxicokinetics in rats	~ 10 mg/kg/day for 5 days	Anatase and rutile (6–90 nm)	Oral particle bioavailability of ~ 0.02%	None	[67]
Toxicokinetics in rats	Up to 2 mg/kg/day for 5 days	Anatase (20–60 nm)	Increased Ti concentrations in spleen and ovaries	Altered testosterone levels, histopathologic findings in thyroids	[68]
Toxicokinetics in rats	Up to 30 mg/kg/day for 7 days	Anatase and rutile (primary sizes not specified)	None detected	None	[37]
Toxicokinetics in rats	12.5 mg/kg/day for 10 days	Rutile (500 nm)	Detection of particles in GALT, lymph nodes and liver	None	[66]
Subacute toxicity in mice	Up to 500 mg/kg/day for 5 days	Anatase/rutile (46 nm)	Not examined	Histopathologic findings in gut mucosa	[134]
Subacute toxicity in mice	Up to 100 mg/kg/day for 14 days	Anatase (20–50 nm)	Not examined	Histopathologic findings in liver	[83]
Subacute toxicity in mice	150 mg/kg/day for 14 days	Anatase (21 nm)	Not examined	Histopathologic findings in liver	[84]
Subacute toxicity in rats	300 mg/kg/day for 14 days	Structure not specified (50–100 nm)	Not examined	Histopathologic findings in liver	[85]
Subacute toxicity in rats	24,000 mg/kg/day for 28 days	Rutile (173 nm)	Detection of particles in GALT	None	[82]
Subacute toxicity in rats	Up to 200 mg/kg/day for 30 days	Anatase (75 nm)	Not examined	Histopathologic findings in liver	[88]
Subchronic toxicity in mice	Up to 250 mg/kg/day for 42 days	Anatase (25 nm)	Not examined	Increased sperm abnormalities	[86]
Subchronic toxicity in mice	64 mg/kg/day for 196 days	Anatase (18 and 120 nm)	~ 0.15 µg/ml Ti in whole blood	Histopathologic findings in liver, kidney, spleen and pancreas	[65]
Subchronic toxicity in rats	Up to 1000 mg/kg/day for 90 days	Coated rutile (145 nm)	Detection of particles in GALT	None	[82]
Subchronic toxicity in rats	Up to 1042 mg/kg/day for 90 days	Anatase/rutile (26 nm)	Marginally higher Ti blood levels in males	None	[39]
Subchronic toxicity in rats	Up to 50 mg/kg/day for 30 and 90 days	Anatase (24 nm)	None	Altered serum enzyme levels	[87]
Carcinogenicity in mice	Up to 8350 mg/kg/day for 2 years	Anatase (pigment-grade)	Not examined	Lower survival, hepatocellular carcinomas	[112]
Carcinogenicity in rats	Up to 2900 mg/kg/day for 2 years	Anatase (pigment-grade)	Not examined	Hyperplastic bile ducts, thyroid carcinomas	[112]
Reproductive toxicity in rats	Up to 1000 mg/kg/day in gestation	Anatase and/or rutile (43–213 nm)	Not examined	None	[114]
Reproductive toxicity in rats	100 mg/kg/day in gestation	Anatase (10 nm)	Increased Ti content in hippocampus	Impaired learning and memory	[116]
Acute colitis model in mice	Up to 500 mg/kg/day for 7 days	Rutile (30–50 nm)	Not examined	Histopathologic findings in gut mucosa	[124]
Colon cancer model in rats	Up to 10 mg/kg/day for up to 100 days	Anatase/rutile (22 and 118 nm)	Detection of particles in GALT and liver	Histopathologic findings in gut mucosa	[135]

In an earlier study, Sprague–Dawley rats were treated with rutile particles (mean size of 500 nm) by oral gavage at a dose of 12.5 mg/kg body weight per day [66]. SEM analysis and histologic examination of tissues after 10 days of dosing revealed the presence of TiO_2_ particles in the GALT and mesenteric lymph nodes and even demonstrated some translocation to sinusoidal cells of the liver. The Ti content of the aforementioned tissues was demonstrated by ICP-atomic emission spectroscopy and quantitative estimates suggested that approximately 6.5% of TiO_2_ particles were absorbed. However, the authors did not consider the background Ti content in their calculations, likely resulting in an overestimation of systemic retention. In another study, Sprague–Dawley rats fed with Ti-free diet received by gavage a single oral dose of TiO_2_ (5 mg/kg) in the form of nano- or micron-sized particles with mean diameters ranging from 40 nm to 5 µm [40]. These particles consisted of anatase or rutile. Ti levels were measured by ICP-mass spectrometry in the feces, blood and urine at different times after administration and no Ti translocation from the gastrointestinal tract into blood or urine was observed. The animals were sacrificed 4 days after administration for tissue analysis, but Ti concentrations in liver, spleen and kidney remained at control levels. In a subchronic study, TiO_2_ particles consisting of 80% anatase and 20% rutile (mean size of 26 nm) were administered orally to Sprague–Dawley rats at daily doses of up to 1042 mg/kg body weight for 90 days [39]. Upon analysis by ICP-mass spectrometry, no increased Ti levels were detected in liver, spleen, kidney and brain tissues even in the group of animals receiving the highest dose, thus indicating a very low oral bioavailability. In the blood taken at necropsy, the high background Ti concentration of ~ 0.4 µg/g was minimally increased, but only in males of the 521 and 1042-mg/kg groups.

Similar results were obtained from a study performed with differentially sized TiO_2_ particles provided by the Joint Research Center (JRC) Nanomaterials Repository. These reference materials consist of anatase or rutile with mean particle sizes ranging from 6 to 90 nm. Wistar rats were administered these particles by oral gavage at a daily dose of around ~ 10 mg/kg body weight for 5 consecutive days [67]. The ICP-mass spectrometry analysis of liver, spleen and mesenteric lymph nodes performed 24 h after the last exposure revealed low Ti levels exceeding the limit of detection of 30 ng/g only occasionally. In a few TiO_2_-exposed animals, there was a detectable but very slight increment of Ti in liver, spleen or the mesenteric lymph nodes. On the basis of these findings, the fraction of TiO_2_ particles absorbed after repeated oral administration was estimated to be maximally 0.02% by weight. Upon intravenous application, the same particles were predominantly retained in the liver, and the subsequent analysis of animals sacrificed at different times after injection reveled long half-lives of up to 650 days in the liver and spleen. It is therefore possible that even a limited systemic absorption from the gastrointestinal tract in combination with slow elimination might potentially result in tissue accumulation.

In a short-term exposure test, anatase particles (primary size of 20–60 nm) were administered by oral gavage to Sprague–Dawley rats at doses of up to 2 mg/kg body weight per day for five consecutive days [68]. ICP-mass spectrometry measurements revealed a slight but statistically significant increase of Ti concentrations relative to untreated controls not only in the spleen but surprisingly also in the ovaries of animals exposed at the higher dose. The penetration of TiO_2_ into the spleen (but not into the ovaries) was confirmed by single-particle ICP-mass spectrometry and SEM analysis of tissue homogenates, thus demonstrating the presence of particle aggregates/agglomerates.

Taken together, the above reports indicate a size-dependent biokinetic behavior with low systemic absorption of orally administered fine-sized TiO_2_ particles displaying primary sizes > 100 nm. This conclusion is confirmed by a toxicokinetic study in Sprague–Dawley rats carried out according to the OECD test guideline 417, which failed to detect any systemic uptake of pigment-grade rutile and anatase particles [37]. Although some penetration may take place across and between enterocytes, the observed intestinal uptake of nano-sized TiO_2_ particles occurs primarily through the GALT as the port of entry [16, 53, 69]. Thus, a unique feature of the gastrointestinal exposure is that a fraction of TiO_2_ particles is retained in the GALT from where the particles may reach the blood presumably through the lymphatic thoracic duct. There is finally only slow elimination of these particles from internal organs (with estimated half-lives of up 650 days), indicating the potential for persistence and accumulation after repeated uptake.

### Oral bioavailability in humans

Studies in adult human subjects highlight a low but detectable oral bioavailability. Male volunteers ingested anatase particles at doses of 23 and 46 mg in gelatin capsules (mean particle size of 160 nm) or as a powder (mean particle size of 380 nm) [70]. Pretreatment blood Ti levels, measured by ICP-atomic emission spectroscopy, ranged between 0.007 and 0.02 µg/ml. After TiO_2_ administration, blood was obtained at different times over 24 h. Around 8–12 h after the intake of 160-nm anatase at the dose of 23 mg (~ 0.4 mg/kg body weight), peak Ti concentrations in the blood reached 0.04–0.05 µg/ml in the five volunteers. The highest Ti concentration of 109.9 µg/l was detected in the blood of one volunteer 8 h after ingesting 46 mg (~ 0.75 mg/kg body weight) of 160-nm anatase. Administration of 380-nm anatase in the same amounts yielded lower blood concentrations. In another human study, nine volunteers received a 5-mg/kg single oral dose of different TiO_2_ particles, i.e., anatase with a size of 15 nm, rutile with a size of ~ 100 nm and another rutile in the micron-scale range, dispersed in water [71]. The ICP-mass spectromety analysis of blood collected over a 4-day period, starting 24 h before dosing and ending 3 days post-dose, revealed that essentially none of the administered particles were systemically absorbed. The background Ti concentration in the blood was 0.014 µg/ml. A further study involved seven volunteers who ingested a single 100-mg dose of anatase (particle size of 260 nm) in the form of gelatin capsules [72]. The particles were subsequently identified in the blood of by dark field microscopy and the presence of Ti was confirmed by ICP-mass spectrometry. A peak Ti blood concentration of ~ 10 µg/ml (against a background of ~ 1.5 µg/ml) was detected at 6 h after ingestion of the test item. Thus, this report presented by Pele et al. [72] not only confirms the earlier finding of Böckmann et al. [70] in revealing a peak of Ti in the blood after oral intake of TiO_2_, but also demonstrates that at least part of this Ti appears in the blood as whole particles. There is an intriguing difference between the background Ti concentration of whole blood in most rodent studies (0.05 µg/ml or higher) and the lower levels observed in human blood (0.007–0.02 µg/ml).

### Impact of the particle corona

A critical aspect that has not yet been investigated with regard to bioavailability and systemic distribution is the effect of bound biomolecules that alter surface properties [73–75]. In fact, small particles avidly and rapidly adsorb on their surface macromolecules including proteins that modify key characteristics like their overall size, aggregation state, bioavailability, tissue distribution and bioaccumulation. The term “corona” was introduced to describe the simultaneous attachment of multiple macromolecules from a physiologic environment to the surface of nanoparticles [76, 77]. For example, TiO_2_ particles incubated in a simulated intestinal digestion juice form a corona of bile acids and proteins [78]. Also, TiO_2_ nanoparticles incubated in blood plasma are readily decorated with a layer of proteins like apolipoprotein A-1, complement factors and immunoglobulins [73–75]. Thus, each biologic compartment has its own set of macromolecules that interact with outer particle surfaces. Although not explicitly tested, it can be assumed that TiO_2_ adopts distinctive corona compositions in the context of each different food matrix in which this material is incorporated as additive [79]. Also, any given corona configuration is expected to progressively change upon oral ingestion, as the particles and surrounding food constituents move through saliva in the mouth to the gastric and intestinal fluids [80]. Presumably, the corona composition further changes if the particles move from the intestinal lumen to the central blood compartment and internal organs. A potential effect of this continuously changing corona is to modify key surface properties, which could mediate particle transfer across biological barriers and their uptake into cells including for example macrophages, dendritic cells or hepatocytes, thus influencing bioavailability and tissue permeability [81]. Importantly, the extent of macromolecular interactions and composition of the resulting corona depends on both the surface chemistry of the particles and their exact diameter. At the outer interface of nano-sized particles, for example, a highly curved surface increases the deflection angle between absorbed macromolecules, possibly leading to a higher density of such macromolecules in the corona of smaller nanoparticles compared to the corona of larger particles [73–75]. Therefore, nanomaterial characteristics like surface chemistry and outer curvature determine the corona composition and these considerations imply that uncoated *versus* coated or small *versus* large particles exert fundamentally different biological effects as a consequence of their distinct corona composition.

### Acute toxicity

An overview of available toxicity studies is shown in Table 1. With the exception of genotoxicity tests, only the oral route of exposure is considered relevant for the risk assessment of TiO_2_ as food additive. No mortality or adverse signs resulted from an acute exposure by single oral gavage administrations of TiO_2_ particles (crystal structure not specified) according to the OECD test guideline 420. A suspension of the test material (mean particle size of 155 nm) was administered to male and female CD-1 mice at the dose of 5000 mg per kg body weight [42]. The same dose of nano-sized TiO_2_ particles (diameters of 25 and 80 nm) resulted in an increased liver weight. There were also histopathologic findings in the brain (fatty degeneration of hippocampal regions), in the liver (hydropic degeneration around the central vein) and kidney (glomerular swelling). These organ damages were reportedly more serious in the animals treated with the 80-nm particles, which is consistent with higher Ti tissue levels achieved by administration of these medium-sized test items compared to the 25 and 155-nm counterparts (see section on oral bioavailability above). No statistical analysis supported the causal relationship between histopathologic findings and TiO_2_ treatments. For an acute oral toxicity study in Crl: CD(SD) rats according to OECD guideline 425, alumina- and silica-coated particles with a primary size of 73 nm and a crystalline composition of 79% rutile/21% anatase were administered by gavage at doses of up to 5000 mg/kg body weight [82]. These treatments with surface-coated particles failed to elicit mortality, biologically relevant body weight changes, clinical signs (except grey-colored feces) or gross organ lesions.

### Subacute and subchronic toxicity

A subacute exposure was carried out in male albino mice with anatase particles displaying a primary size of 20–50 nm (see Table 1 for an overview of oral toxicity studies). Daily doses of 10, 50 and 100 mg/kg body weight were applied for 14 consecutive days [83]. At the highest dose, this treatment induced a statistically significant increase of liver weight and histologic changes including a recruitment of mononuclear cells to the vicinity of sinusoids accompanied by angiectasis (dilated sinusoidal spaces filled with blood cells). Another 14-day oral exposure study was carried out in male albino mice with anatase particles displaying a mean diameter of 21 nm [84]. The particles were administered daily at 150 mg/kg body weight, leading to statistically significantly higher liver weights as well as significantly increased serum levels of the liver enzymes alanine aminotransferase (ALT) and aspartate aminotransferase (AST). The authors also reported histopathologic changes in the liver (focal degeneration of hepatocytes with mononuclear cell infiltration) supporting the hypothesis that the tested nanoparticles cause liver injury. Swelling and vacuolization of hepatocytes as well as infiltration of inflammatory cells were additionally detected in the liver of Wistar rats treated daily for 14 consecutive days by oral gavage with 300 mg/kg TiO_2_ particles (composition not specified, primary size of 50–100 nm) [85]. These adverse hepatocellular effects were supported by a statistically significant increase of ALT, AST and alkaline phosphatase serum activity in treated animals compared to vehicle controls.

A 28-day study was carried out in line with OECD test guideline 407 using rutile particles with a mean size of 173 nm. This material was administered by oral gavage to 8-week old male Cr: CD(SD) rats at daily doses of 24,000 mg/kg body weight [82]. One rat each from the control and test group died during the dosing period due to accidental perforation of the esophagus. There were, however, no test item-related effects on mortality, food intake, body weight, clinical signs, hematology, serum clinical chemistry, hematology, organ weights, gross pathology or histopathology. Brown granular aggregates or clumps, likely indicative for the presence of TiO_2_, were seen upon hematoxylin and eosin staining in sections of the intestinal mucosa and draining lymphoid tissue, but without overt cellular reactions. These microscopic findings related to the presence of test particles in the GALT were not considered to be adverse.

An oral subchronic (90-day) toxicity study was performed in line with OECD test guideline 408 using rutile particles with a mean diameter of 145 nm. The particle surface was alumina-coated. This material was administered to groups of 8-week old Cr: CD(SD) rats by daily gavage doses of up to 1000 mg/kg body weight [82]. There were no treatment-related effects on survival, food intake, body weight, clinical signs, hematology, clinical chemistry, hematology, organ weights, gross pathology or histopathology. Test material-related findings were limited to microscopic observations consistent with the oral route of uptake. In particular, granular aggregates or clumps, indicating the presence of TiO_2_, were seen in the intestinal mucosa and the draining lymphoid tissue, without tissue reactions. Again, these findings related to the presence of TiO_2_ particles in the GALT were not considered adverse. There is also a 28-week study with CD-1 mice exposed orally at 64 mg/kg/day to anatase particles with mean diameters of 18 and 120 nm [65]. The authors reported histopathologic findings such as tissue fractures in the liver, glomerular atrophy in kidneys and islet hyalinization in the pancreas induced by 18-nm particles but not upon treatment with 120-nm particles, consistent with the missing systemic retention of the latter (see section on oral bioavailability above).

In summary, these oral toxicity studies in rodents reveal major uncertainties limiting their predictive value for the risk assessment of human dietary exposure. Many reports are based on a small number of animals per treatment group, involve an unusual or inadequate design or lack statistical analyses. Some studies used insufficiently characterized particles with regard to composition, possible contaminants, impurities or physico-chemical properties, and most reports failed to monitor particle size distributions. Single-dose [42] or repeated-dose oral exposures [83–85] point to liver toxicity as a common endpoint following gastrointestinal absorption of nano-sized TiO_2_ particles with mean diameters below 100 nm. This endpoint involving liver toxicity is not seen after oral administration of TiO_2_ particles with mean diameter above 100 nm [82]. Further adverse outcomes in oral toxicity studies were reported by the Medical College of Soochow University (Suzhou, China). These studies tested TiO_2_ nanomaterials synthesized by technical procedures that are not consistent with commercial products in the food sector. Three of these studies indicating toxicity in animals were withdrawn by journal editors due to inadequate statistical analyses. As already pointed out [13, 29], the same methodological deficits are also found in other publications from the same group such that their reports were not further considered.

### Oral toxicity in young animals

In view of the higher exposure of children relative to adults (see section on human exposure), it is also appropriate to screen the literature for oral toxicity studies carried out in young laboratory animals. A seminal report involved pubertal male mice aged 4 weeks at the beginning of exposure. Anatase particles with a mean diameter of 25 nm were administered orally at daily doses of 10, 50 and 250 mg/kg body weight for 42 consecutive days [86]. The analysis of epididymal sperm at the end of the exposure period revealed a statistically significant and dose-dependent increment of morphologic abnormalities. Although no changes in sperm number were detected, the fraction of sperm cells displaying abnormalities increased from ~ 13% in controls to ~ 23% in the 50-mg/kg group and ~ 29% in the 250-mg/kg group. These effects on spermatogenesis were associated, in the medium- and high-dose groups, with a reduction of serum testosterone levels, decreased layers of spermatogenic cells and an increased appearance of vacuoles in the seminiferous tubules. No statistical evaluation of these histologic findings was given. The testosterone reduction described in the young mice of this report is contrasted by another study using 9–10-week old rats, described in more detail in the section on reproductive toxicity below, where anatase particles (primary size of 20–60 nm) given by the oral route were associated with increased serum testosterone levels in males, whereas the concentration of this same hormone was reduced in females [68]. Another study focused on cardiac toxicity in young rats (4 weeks old at the beginning of the study) following 30 and 90 days of an oral exposure to anatase particles (mean size of 24 nm) at 2, 10 and 50 mg/kg body weight per day [87]. The authors report changes in some biochemical endpoints like decreased serum lactate dehydrogenase, hydroxybutyrate dehydrogenase and creatine kinase activity in the high-dose group, but this study did not reveal any toxicologically relevant effects. A comparison between rats aged 4 weeks and rats aged 9 weeks at the start of the study was carried out to test the susceptibility to anatase particles with a mean size of 75 nm [88]. Sprague–Dawley rats were administered daily doses at 10, 50 or 200 mg/kg body weight for 30 consecutive days. Histologic examinations of the organs after the treatment period revealed changes in the liver described by the authors as hepatic cord disarray, perilobular cell swelling, vacuolization and hydropic degeneration in both the 50 and 200-mg/kg dose groups, but only in young animals. These alterations were accompanied by a statistically significant rise of serum bilirubin at the anatase dose of 200 mg/kg. In adult rats, less serious infiltrations of inflammatory cells in the liver parenchyma were seen at 10 and 50 mg/kg (but not at 200 mg/kg) and considered to represent background liver lesions frequently observed in rats.

In summary, these few studies on the reaction of differentially aged rodents suggest the possibility that young animals may be more susceptible than adults to developing adverse effects upon oral exposure to TiO_2_ particles.

### Genotoxicity

TiO_2_ particles with varying composition (anatase, rutile or mixtures of these two polymorphs) and different sizes were probed for mutagenicity in the canonical reverse mutation assay with bacteria (Ames test), usually at concentrations of up to 5–10 mg per standard plate. In all cases, the tested particles failed to elicit mutations in the absence or in the presence of rat liver microsomes mediating metabolic activation (see for example [89–93]). However, the Ames test is not considered suitable for this purpose due to the presumed inability of bacterial cells, conferred by their rigid cell wall, to take up the particles.

Conflicting findings were reported from in vitro tests carried out in rodent or human cells and aimed at the detection of DNA strand breaks, point mutations, deletions, chromosomal aberrations, micronuclei or sister chromatid exchanges. Mammalian cell-based genotoxicity assays yielded both positive and negative outcomes when used to test TiO_2_ particles. These methods include, in particular, single-cell electrophoresis (comet assays) as well as reporter gene, micronuclei, chromosomal aberration and sister chromatid exchange assays (see for example [83, 91, 92, 94–107]). The interpretation of findings is uncertain because the effects, if any, may be secondary to cytotoxic or apoptotic reactions elicited by incubation of rodent or human cells with high concentrations of the test material.

For the evaluation of in vivo genotoxicity, it is important to consider that, in view of the low absorption of TiO_2_ particles after oral uptake (see section on oral bioavailability), sufficient systemic exposure can only be achieved by parenteral administration. For example, B6C3F1 mice were subjected to daily intravenous injections of anatase particles (mean size of 10 nm) at doses ranging from 0.5 to 50 mg/kg body weight, for 3 consecutive days. Genotoxicity was assessed by determining the micronuclei in reticulocytes and the frequency of mutations in the X chromosome-linked phosphatidylinositol glycan complementation group A (Pig-A) gene in peripheral blood cells [108]. This study concluded that anatase nanoparticles reaching the bone marrow cause considerable cytotoxicity but without inducing direct genotoxic effects. Transgenic C57Bl/6 mice harboring the bacterial *lacZ* reporter gene were injected intravenously with anatase particles (mean size of 22 nm) obtained from the JRC Nanomaterials Repository, at daily doses of up to 30 mg/kg body weight on 2 consecutive days [109]. No genotoxic effects were detected by scoring the frequency of micronuclei in reticulocytes of the peripheral blood and by monitoring *lacZ* mutations as well as DNA strand breaks (by comet assay) in liver and spleen cells. Anatase/rutile particles (primary size of 21 nm) were injected intravenously to Wistar rats at a single dose of 5 mg/kg body weight [110]. No genotoxicity was subsequently detected by analyzing bone marrow cells in the comet assay. A threefold increase in micronucleated cells was detected 1 h after the injection when polychromatic erythrocytes were stained with the conventional May-Grunwald-Giemsa reagents, but not in concomitant stains with acridine orange. Although not recommended by the relevant OECD test guideline 474, other authors selected the intraperitoneal route to expose rats with anatase/rutile particles displaying a primary size of 45 nm. The daily doses were 500–2000 mg/kg body weight applied for 5 consecutive days [111]. The animals were sacrificed 24 h after the last treatment and genotoxic effects were evaluated by counting the frequency of micronuclei in polychromated erythrocytes of the bone marrow, and by monitoring the appearance of DNA strand breaks when subjecting bone marrow, brain and liver cells to comet assays. These experiments revealed a statistically significant and dose-dependent increase in micronuclei and DNA strand breaks in response to the TiO_2_ treatment. However, the report lacks an assessment of cytotoxicity in the tested tissues raising the possibility that the genotoxic effects might be secondary to particle-induced cell death. The above considerations lead to the conclusion that there is no solid evidence for anatase/rutile particles being genotoxic.

### Carcinogenicity

Long-term (2-year) carcinogenicity studies were carried out in both B6C3F1 mice and Fisher 344 rats using, as the test material, pigment-grade anatase particles designated Unitane^®^ 0–220 [112]. The size of these test particles is not specified but it can be assumed from their optical properties that they have a mean diameter in the 200–300 nm range conferring a white color. The test material was included in the diet of mice (groups of 50 males and 50 females) at daily doses of 3250 and 6500 mg/kg body weight (for male animals) and 4175 and 8350 mg/kg (for females). The particle-supplemented feed was not examined for possible nutritional imbalances. With the exception of white feces, there were no clinical signs related to TiO_2_ administrations. The test item did not affect the survival of male mice but, in the females of the high-dose group, only 66% survival was reported until the end of the 104-week study compared to 90% survival in controls. No accompanying effects were reported that would explain this gender-specific difference. All surviving animals were sacrificed after 103 weeks for comprehensive macroscopic and microscopic inspection for neoplasms. There was a dose-dependent increase in the incidence of hepatocellular carcinomas in males (17% of animals in the control group, 19% in the low-dose group and 29% in the high-dose group), although the test laboratory noted that this higher occurrence of liver cancer remained within the range of historical reference controls. The study authors, therefore, concluded that the oral TiO_2_ administration is not carcinogenic in the tested mice strain.

In rats, the same Unitane^®^ 0–220 particles were tested by dietary administration to groups of 50 males and 50 females at daily doses of 1125 and 2250 mg/kg body weight (for males) and 1450 and 2900 mg/kg (for females). Again, the particle-supplemented feed was not examined for possible nutritional imbalances. With the exception of white feces, there were no clinical signs related to TiO_2_ exposure. The test substance had no effect on survival. After 103 weeks, the organs of surviving animals were subjected to macro- and microscopic analyses. There was an increased frequency of hyperplastic bile ducts in males of both the low- and high-dose groups. Tumor incidences in the treatment groups were reportedly not higher than in controls. However, in females the combined incidence of c-cell adenomas and carcinomas of the thyroid was substantially increased from 2% in controls to 14% in the high-dose group. No such adenomas or carcinomas were detected in the low-dose group. Although statistically significant by the Cochran–Armitage test (*P* = 0.013) and the Fisher exact test (*P* = 0.042), this increased neoplastic incidence was dismissed by introducing a Bonferroni correction for multiple comparisons. Irrespective of such statistical considerations, the emergence of thyroid tumors in rats needs careful considerations due to the questionable relevance of this finding for humans [113].

### Reproductive toxicity

A pivotal prenatal developmental study evaluated three pigment-grade (pg-1, pg-2 and pg-3) and three ultrafine (uf-1, uf-2 and uf-3) anatase and/or rutile particles given to pregnant rats following the OECD test guideline 414. The primary particle size was 153–213 nm for the pigment-grade material and 43–47 nm for the ultrafine material. These test substances were administered to Crl:CD(SD) rats by oral gavage on gestation days 6–20. Also, pregnant Wistar rats were exposed to TiO_2_ particles (uf-2, pg-2 and pg-3) by oral gavage on gestation days 5–19. The dose levels in both rat strains were 100, 300 and 1000 mg/kg body weight per day [114]. At the end of each exposure period, the rats were sacrificed for caesarean sections and examination of the dam and fetuses. As the only finding, at 1000 mg/kg per day, the uf-1 particles led to an increased fetal sex ratio (males/females) from 0.46 in the controls to 0.60 in the treatment group. The range of the test facility historical control for this parameter was 0.43–0.53. Because the sex ratio is determined by events that occur around conception well before the start of the treatment on gestation day 6, the authors concluded that this finding was not test item-related. These results are confirmed by a reproduction study according to OECD test guideline 421, which involved Sprague–Dawley rats dosed with daily oral gavages of pigment-grade TiO_2_ at 1000 mg/kg body weight. The males were administered the test substance for 40 days (beginning from 2 weeks before the mating period) and females were treated for 2 weeks before mating, during the gestation period and until day 4 after delivery. Also this study did not reveal any reproductive or developmental toxicity [115].

Other reports ascribe adverse reproductive effects to orally administered TiO_2_ particles but, unfortunately, these further experiments in rodents were not carried out following standardized procedures. Undoubtedly, these studies raise uncertainties but their impact on hazard identification and characterization is still limited in view of the small number of animals per treatment group, inappropriate study design or insufficient statistical analyses. In a study already mentioned in the preceding sections, anatase particles (primary size of 20–60 nm) were given to Sprague–Dawley rats by the oral route at doses of up to 2 mg/kg body weight per day for 5 consecutive days [68]. This oral exposure was associated with increased serum testosterone levels in males, whereas the concentration of this hormone was reduced in females. The authors also reported histologic changes in the thyroid (desquamation into follicular lumen, follicles with irregular shape, smaller colloid space) after oral treatments at 1 or 2 mg/kg body weight per day for 5 days.

Neurodevelopmental consequences of a TiO_2_ exposure were suggested by a study in which pregnant Wistar rats were treated by oral gavage with anatase particles (primary size of 10 nm) at 100 mg/kg body weight [116]. This dose was applied daily from gestation day 2 to gestation day 21. Two male pups from each litter were sacrificed for the examination of brains immediately after birth. The Ti content in the hippocampus of the pups in the test group was increased to a statistically significant degree. Concomitantly, the authors observed that expression of the cell proliferation marker Ki67 is reduced in that brain region of treated animals relative to controls. On post-natal day 60, the learning and memory capacity was tested in randomly selected male pups by means of the passive avoidance and Morris water maze test, and was found to be impaired in the treatment group relative to controls.

### Local effects in the gastrointestinal tract

Depending on the identified hazards, the usual risk characterization may need the assessment of additional endpoints that are not routinely inspected in the toxicological evaluation of most chemicals. One question is whether TiO_2_ particles may influence directly the bacterial community in the gut lumen. When tested in vitro using anaerobic reactors, which provide an intestinal microbiome surrogate, food-grade anatase has been shown to cause marginal shifts in bacterial populations, for example by slightly reducing the abundance of *Bacteroides ovatus* in favor of a *Clostridium* species as seen at particle concentrations of 100 µg/ml or higher [117, 118]. The biological relevance of these observations needs to be ascertained but it appears so far that TiO_2_ particles do not exert major effects on the human gut microbiota at realistic concentrations.

Another potential target of nanomaterials is the intestinal surface under the surveillance of dendritic cells that act as first-line sentinels of foreign materials by filtering out a volume of up to 1500 µm^3^, which equals their own cell volume, per hour [119]. Unlike other antigen-presenting cells, dendritic cells constitutively express class II major histocompatibility complexes and, in response to pathogen recognition, display co-stimulatory surface glycoproteins and secrete inflammatory cytokines. By these combined features, dendritic cells constitute key activators of both the innate and adaptive immune system [120, 121]. It is not surprising to find that, based on their function in sampling their environment for intruding insults, dendritic cells are also able to capture nanoparticles in an efficient manner [122]. It was shown in vitro that endotoxin-activated dendritic cells release the potent pro-inflammatory cytokine interleukin-1β (IL-1β) upon incubation with nano-sized anatase/rutile particles [123]. By activation of the inflammasome, leading to IL-1β secretion, gavage applications of rutile particles (30–50 nm) at a daily dose of 50 mg/kg body weight aggravate macro- and microscopic signs of acute colitis induced in C57BL/6 mice by repeated exposures to dextran sulfate sodium (DSS) given in the drinking water [124]. In addition, a nano-Trojan horse hypothesis has been proposed due to the enhanced adsorptive surface property of nanoparticles and, hence, their potential to carry harmful substances as their cargo [125]. A relevant pro-inflammatory cargo of dietary inorganic particles like those containing TiO_2_ consists of bacterial fragments such as lipopolysaccharides (LPS) or peptidoglycan [126–128]. In this context, pigment-grade TiO_2_ particles have been shown to stimulate secretion of IL-1β from macrophages isolated from mice carrying a mutation in the *nucleotide*-*binding oligomerisation domain*-*containing 2* (*NOD2*) gene [129], a mutation that confers an increased risk for inflammatory bowel disease (IBD) [130, 131]. It is, therefore, possible that the binding of luminal antigens or adjuvants to TiO_2_ particles could aid their delivery to inflammatory cells of the gastrointestinal tract and contribute to the pathogenesis of IBD in susceptible individuals. Conversely, dietary nanoparticles by adsorptive sequestration on their surface may negatively influence the bioavailability of iron, zinc and fatty acids [132, 133].

Reactions of the gastric mucosa were examined after oral treatment of Swiss Webster mice with nanoparticles (mean size of 46 nm) composed of rutile (77%) and anatase (22%). The TiO_2_ nanoparticles were dispersed in water and administered at daily doses of 5, 50 and 500 mg/kg body weight for 5 consecutive days. The animals were sacrificed 24 h, 1 or 2 weeks after the last treatment. Analysis of the gastric epithelium by ICP-mass spectrometry revealed a dose-dependent increase of Ti levels associated with histopathologic effects like submucosal edema, necrosis of epithelial cells and ulcerations [134]. The severity of these histopathologic findings increased with the dose. Another approach to this problem made use of a colorectal cancer model to test the ability of food-grade TiO_2_ particles to accelerate intestinal tumor progression. This murine model involves the intraperitoneal injection of the tumor initiator azoxymethane (AOM) combined with repeated exposures to the pro-inflammatory agent DSS in drinking water. BALB/c mice were treated with the AOM/DSS protocol, with intragastric gavage of TiO_2_ particles at 5 mg/kg body weight per day alone, or with a combination of AOM/DSS and TiO_2_ particles [18]. The primary size of these particles is not specified but TEM analyses revealed aggregates/agglomerates with diameters ranging from 50 to 600 nm. The particle administrations took place during 5 days per week for 10 weeks. Necropsies carried out after 11 weeks revealed that the combination of AOM/DSS with TiO_2_ particles increased the expression of markers of tumor progression (COX2, Ki67 and β-catenin) in the epithelium of the colon, whereas TiO_2_ alone did not induce such expression changes. Moreover, the authors observed that the tested food-grade TiO_2_ particles, even in the absence of any tumor initiator/promoter, reduced the density of protective mucin-producing goblet cells detected by alcian blue staining.

The consequences of an oral exposure to food-grade TiO_2_ on the intestinal mucosa was further investigated in male Wistar rats. Two types of anatase/rutile materials were tested: commercial E 171 (primary particle size of 118 nm) and, as a reference, nanoparticles from the JRC Nanomaterials Repository (primary size of 22 nm). In a first experiment, these test materials were given by daily gavage administrations at 10 mg/kg body weight for 7 days in the absence of any additional treatment. In a second experiment, the animals were pretreated with the tumor initiator 1,2-dimethylhydrazine (DMH) followed by exposure to E 171 at daily doses of 0.2 and 10 mg/kg body weight for 100 days through the drinking water. Another experimental group was exposed to E 171 for 100 days without DMH pretreatment [135]. Interestingly, exposure to E 171 in these experiments led to the internalization of light-diffracting particles not only in cells of the Peyer’s patches but, to a minor extent, also in the colonic mucosa and liver, particularly in proximity of the portal vein sinus. An accumulation of both E 171 and TiO_2_ nanoparticles in the Peyer’s patches of treated rats was confirmed by secondary ion mass spectrometry. Effects on the lymphoid tissue included an increased number of dendritic cells in Peyer’s patches and a higher capacity of spleen cells to secrete cytokines like interferon-γ and IL-17, possibly dysregulating immune responses. In the carcinogenesis model, exposure to E 171 at 10 mg/kg per day led to a statistically significant increase of aberrant crypt foci (defined as abnormal tube-like glands in the lining of the colonic mucosa) regardless of whether the animals were pretreated with DMH or not. Based on these findings, the authors concluded that food-grade TiO_2_ particles induce a low-grade local inflammation in the mucosa that has the potential to initiate preneoplastic lesions in the colonic mucosa. An important caveat in this interpretation of the reported findings is that the relevance of abnormal crypt foci as an early precursor of colorectal cancer is controversially discussed.

## Conclusions

The Joint FAO/WHO Expert Committee on Food Additives considered unnecessary the establishment of an acceptable daily intake (ADI) for TiO_2_ additives in food [136]. This decision was taken on the basis of the low solubility, poor absorption into internal organs like liver and the absence of acute toxic effects. Many reports presented in the context of this review suggest that, contrary to the assumptions made 50 years ago, food-grade TiO_2_ particles are not totally inert upon oral intake. The observation that TiO_2_ particles cause at least some adverse reactions in experimental animals is disturbing because this material, including its unavoidable nano-scale byproducts, has been in use as food additive since 1966. The now available literature reveals data gaps and uncertainties that should be addressed before declaring food-grade TiO_2_ particles as generally safe. A non-exhaustive list of such data gaps und uncertainties comprises the following.

- Although there is only limited absorption from the gastrointestinal tract, toxicokinetic experiments in rats revealed very long tissue half-lives for TiO_2_ nanoparticles in internal organs [67]. This observation indicates that there is the potential for a slow but continued accumulation of particles upon lifelong exposure.
- The public literature offers only one subchronic oral toxicity study in rodents carried out following internationally recognized OECD test guidelines [42]. This study was based on the daily administration of food-grade TiO_2_ consisting of coated rutile particles. There were no test item-related adverse effects, yielding a no observed effect level (NOEL) of 1000 mg/kg body weight per day, which corresponds to the highest dose tested. By applying a default uncertainty factor of 200 (to adjust for inter-species as well as inter-individual variations in sensitivity, and for the extrapolation from subchronic to chronic exposure [137]), this NOEL of 1000 mg/kg/day translates to a tentative safe upper level for the lifetime intake of TiO_2_ particles of 5 mg/kg body weight per day. However, the alumina-coated rutile under scrutiny is not at all representative for the full range of TiO_2_ used in the food sector, which includes anatase or mixed anatase/rutile polymorphs, particularly also in an uncoated form.
- Acute [42], subacute [83–85, 88], subchronic [42] and chronic toxicity studies [112] converge on the liver as a possible target organ for adverse effects after oral TiO_2_ intake (Table 1). The carcinogenicity study in mice revealed an increased incidence of hepatocellular carcinomas in males at 6500 mg/kg/day (but not at 3250 mg/kg/day) compared to controls. The lack of genotoxicity of TiO_2_ particles allows to assume a thresholded mode of liver cancer promotion and, by application of a default uncertainty factor of 100 (to adjust for inter-species and inter-individual variations in sensitivity [137]), the resulting no observed adverse effect level (NOAEL) of 3250 mg/kg/day translates to a tentative safe upper level for the lifetime intake of 3.25 mg/kg body weight per day. The subacute 30-day study by Wang et al. [88] identified a NOAEL for anatase nanoparticles in young animals of 10 mg/kg body weight per day. Assuming a nanoparticle fraction of 4.2% by weight [46], this value of 10 mg/kg corresponds, in terms of food-grade TiO_2_, to a NOAEL of 238.1 mg/kg body weight per day. By applying a default uncertainty factor of 600 (to adjust for inter-species as well as inter-individual variations in sensitivity, and for the extrapolation from 30 days to chronic exposure as proposed by Heringa et al. [29]), the NOAEL of 238.1 mg/kg/day yields a tentative safe upper level for the intake of TiO_2_ particles of 0.40 mg/kg body weight per day.
- One study suggests effects on spermatogenesis resulting in sperm abnormalities upon intragastric gavage administration of anatase nanoparticles for 42 days [86]. This study was carried out in pubertal mice and is, therefore, relevant to address the possibly higher vulnerability of children. The NOAEL, as a nanoparticle dose, was 10 mg/kg body weight per day. Assuming as above a nanoparticle fraction of 4.2% by weight, this 10-mg/kg value corresponds again, in terms of food-grade TiO_2_, to a NOAEL of 238.1 mg/kg body weight per day. By applying a default uncertainty factor of 400 (to adjust for inter-species as well as inter-individual variations in sensitivity, and for the extrapolation from 42 days to chronic exposure as proposed by Heringa et al. [29]), this NOAEL of 238.1 mg/kg/day yields a tentative safe upper level for the intake of TiO_2_ particles of 0.40 mg/kg body weight per day. Another study indicates a penetration of TiO_2_ nanoparticles into the ovaries from the gastrointestinal tract of rats [68]. A further report suggests neurodevelopmental disturbances upon exposure to TiO_2_ nanoparticles during pregnancy in rats [116]. These findings, if confirmed, would raise additional concerns regarding the reproductive safety.
- There is finally only poor understanding of the consequences of the proven uptake of TiO_2_ by reactive GALT cells, possibly triggering inflammatory responses that could favor chronic conditions like IBD, and perhaps leading to initiation, promotion and/or progression of neoplasms in the mucosa of the gastrointestinal tract [18, 134, 135]. Food-borne inorganic particles have been shown to accumulate during lifelong exposure in “pigment cells” of the GALT where the earliest signs of IBD are noted [126, 138, 139]. This is a group of chronic conditions ranging from Crohn’s disease (affecting all segments of the digestive tract) to ulcerative colitis (restricted to the large bowel) [140]. IBD has a multi-factorial etiology with genetic susceptibility, gut microflora composition and a dysfunctional local immune reaction as main drivers [141]. Dietary factors have also been implicated in IBD and several authors raised the concern that inorganic particles in food may contribute to initiating this chronic inflammatory condition by inappropriate stimulation of the innate immune system [55, 56, 142, 143].

In conclusion, the existing toxicity studies cannot completely exclude human health risks from the long-term ingestion of TiO_2_ particles. The above hypothetical upper safe levels for dietary intake (between 0.4 and 5 mg/kg body weight per day) calculated from rodent studies are in no way conclusive and are only meant to illustrate in quantitative terms the wide range of uncertainty in the current risk assessment of this ubiquitous food additive. Especially the estimated consumption by children suggests that, in any case, the dietary exposure to TiO_2_ particles should be reduced to remain, even in a worst-case exposure scenario, below this proposed lowest safety threshold of 0.4 mg/kg daily. Further studies are needed to reduce existing uncertainties.

### Recommendations

The uncertainty emerging in the retrospective assessment of TiO_2_ particles demonstrates the need for a fit-to-purpose data requirement for the future evaluation not only of nano-sized but also of novel submicron-sized particles to be used as food additives. In particular, this review identified the following five main issues related to particles that resist rapid degradation or dissolution under digestive tract conditions:

- To become eligible for the safety assessment, novel particles should first undergo a detailed characterization to provide unambiguous information on their constituents, structure and shape, surface characteristics and coating, average size and size distribution, impurities, aggregation or agglomeration states. All these parameters should be specified with accompanying analytical methods.
- Particle characteristics like size, size distribution, crystalline form, shape and coating are critical determinants of intestinal uptake and adverse reactions (see for example the above sections on particle corona and local gastrointestinal effects). Therefore, there is little opportunity for read-across procedures to assess simultaneously multiple variants of the same particulate material. Instead, we advocate a case-by-case testing of particles with clearly established specifications.
- The potential genotoxicity of nano- and submicron-size particles should be ruled out with mammalian cell models and, pending on the findings, using in vivo genotoxicity tests with proven systemic exposure, following generally recognized OECD guidelines.
- In the absence of any evidence of genotoxicity, the safety testing should proceed with an extended 90-day oral toxicity assay in rodents following OECD test guideline 408 [144], but with additional parameters for the detection of endocrine disruptors as listed in test guideline 407 [145]. This subchronic study should also include the analysis of tissues (including the GALT, mesenteric lymph node, spleen and liver) to examine the degree of systemic particle uptake. Such a toxicokinetic analysis should compare, if necessary using satellite groups of animals, the tissue level of particles at an early and late time of the study to exclude a possible accumulation.
- Additionally, more research is needed to understand the consequences of interactions of indigestible nano- and submicron-sized particles with the GALT and, in particular, with dendritic cells residing in the intestinal mucosa. As outlined in the section on local effects in the gastrointestinal tract, dendritic cells are the first-line sentinels of foreign materials as well as key activators of both the innate and adaptive immune system and, as such, potential triggers of particle-induced chronic inflammatory conditions.

## Abbreviations

ANS: Scientific Panel on Food Additives and Nutrient Sources added to Food
AOM: azoxymethane
DMH: dimethylhydrazine
DSS: dextran sulfate sodium
EFSA: European Food Safety Authority
EU: European Union
GALT: gut-associated lymphoid tissue
IARC: International Agency for Research on Cancer
IBD: inflammatory bowel disease
ICP: inductively coupled plasma
Ig: immunoglobulin
IL: interleukin
JRC: Joint Research Centre
LOD: limit of detection
LPS: lipopolysaccharides
M-cell: microfold cell
NOAEL: no observed adverse effect level
NOEL: no observed effect level
OECD: Organization for Economic Co-operation and Development
ROS: reactive oxygen species
SEM: scanning electron microscopy
TEM: transmission electron microscopy
Ti: titanium
TiO_2_: titanium dioxide
UV: ultraviolet
V: vanadium
